# The gut–liver axis calibrates PEDF production for ISC homeostasis

**DOI:** 10.52601/bpr.2024.240904

**Published:** 2024-06-30

**Authors:** Ying Huang, Xinran Wang, Lulu Sun

**Affiliations:** 1 State Key Laboratory of Female Fertility Promotion, Department of Endocrinology and Metabolism, Peking University Third Hospital, Beijing 100191, China; 2 Institute of Advanced Clinical Medicine, Peking University, Beijing 100191, China

The biliary tract, portal vein, and systemic circulation are the anatomical linkages that enable bidirectional inter-organ communications through the gut–liver axis. (Han *et al.* 2023). Under physiological conditions, the proliferation of intestinal stem cells (ISCs) materializes the self-renewal of the intestinal epithelium for gut barrier integrity (van der Flier and Clevers 2009), and such crosstalk contributes to the repair capacity of the intestinal tract by accommodating the ISCs (Chen *et al.* 2022; Hegyi *et al.* 2018). Nonetheless, in pathological conditions such as inflammatory bowel disease (IBD), the compromised intestinal barrier caused by inflammation increases the translocation of microorganisms (Wigg *et al.* 2001). This microbial-associated molecular pattern (MAMP) affects liver pathology via portal circulation (Gäbele *et al.* 2011). However, it remains to be urgently investigated how such gut–liver reciprocal interflow influences intestinal physiology, particularly the underlying molecular mechanisms concerning ISCs homeostasis. Encouragingly, a recent study by Kim *et al.* revealed that hepatic pigment epithelium-derived factor (PEDF), both as a gatekeeper and self-calibration messenger of the gut–liver axis, could regulate ISC proliferation (Kim *et al.* 2024). This study enriches the critical role of the gut–liver axis in maintaining intestinal physiology and provides potential molecular targets for therapeutic intervention in intestinal inflammation.

Kim *et al.* observed a significant incremental epithelial proliferation in both the small intestine and colon of hepatectomized mice, which could be attributed to hepatokines after transcriptomic analysis of IECs. They then concluded by Gene Ontology analysis, plasma proteomics, combined with published secretomic results from hepatocytes that it could be hypothesized that liver-derived PEDF constraints ISC expansion as the canonical Wnt repressor, which was subsequently validated in *Pedf *^−/−^ mice, *Lgr5*^EGFP^ reporter mice, and liver-specific PEDF deficient mice. To gain deeper insight into the mechanisms underlying PEDF regulating ISC proliferation, Kim *et al.* performed experiments on intestinal organoids *in vitro* to elucidate that PEDF exactly suppresses ISC proliferation by inhibiting the Wnt/β-catenin pathway. Moreover, the authors devised ISCs-specific LRP5 and LRP6 knockout mice to demonstrate that LRP5 and LRP6 are necessary as coreceptors for Wnt ligands to allow PEDF-mediated gut–liver communication for ISC proliferation.

Given the self-renewal devotion of ISCs to gut inflammation, the authors selected dextran sulfate sodium salt (DSS)-induced colitis to explore the role of PEDF in this procedure. The authors found that ISCs proliferation was negatively correlated with hepatic and circulating PEDF during representative stages of DSS colitis in mice, pointing to a conceivable in-between causative link on the gut–liver axis. The authors then compared the changes in hepatocyte transcriptomics during DSS colitis and revealed that Toll-like receptor (TLR) and nuclear factor-κB (NF-κB) signaling pathways were upregulated. Meanwhile, they observed more severe colitis with a targeted deletion of MyD88 in hepatocytes, associated with impaired ISC proliferation, demonstrating that MAMP is critical for hepatic PEDF production. Next, Kim *et al.* aimed to investigate the impact of lipopolysaccharides (LPS), as a typical MAMP, on PEDF during gut inflammation. The researchers employed LPS treatment and elimination of the hepatic LPS sensing receptor (*AlbTlr4*^fl/fl^) to demonstrate that blockade of LPS-TLR4 signaling resulted in dampened peripheral PEDF levels, which facilitated tissue repair and inflammation rehabilitation via restoring ISC proliferation. Furthermore, the researchers were keenly aware that PPARα exhibited the aforementioned fluctuations in PEDF levels during the development of colitis. LPS-mediated extracellular signal-regulated kinase (ERK) activation abrogates the binding of hepatocyte nuclear factor 4 (HNF4) to the Ppara promoter and attenuates PPARα expression, while PPARα promotes PEDF expression by binding to the Pedf promoter. In addition, it was perceived that upregulated PEDF by using fenofibrate (FB), the PPARα agonist, increased the susceptibility of mice to colitis but could be mitigated by PEDF-neutralizing antibodies. Finally, at the clinical level, Kim *et al.* found that IBD patients had reduced PEDF levels in the acute phase and a corresponding negative correlation with circulating LPS and ISC proliferation.

In summary, the work of Kim *et al.* confirms that the gut–liver axis can modulate ISC fitness through PEDF. In physiological terms, PEDF, a soluble liver-derived factor, can confine ISC proliferation, whereas when intestinal inflammation occurs, excess LPS oppresses PEDF expression through the hepatic PPARα signaling pathway and paradoxically promotes ISC proliferation for the repair of damaged tissues. This pioneering study sheds light on a protective negative feedback loop of the gut–liver axis, substantially broadening the comprehension of organ mutual interactions aiding intestinal physiology and inflammation (Fig. 1). This finding also yields new insights for clinical drug development, whereby, for instance, FB as a PPARα agonist may hinder colon tumor progression through the principle of PEDF inhibition of ISC hyperproliferation (Luo *et al.* 2019), while the pro-inflammatory sidelights are based on the impaired ISC repair capacity (Qi *et al.* 2014). It might be useful to develop PPARα treatments with liver specificity or use PEDF-neutralizing antibodies to enhance the utility of the PPARα pathway in the practice. In the research, given that diverse polypeptides of PEDF show different biofunctions (Kim *et al.* 2024), the researchers found that 34-mer could serve as the active fragment of PEDF to exacerbate colitis, which on the one hand, the peptide could not completely replace the PEDF protein due to its instability, while on the other hand, it also offers a certain enlightenment for the more studies of PEDF polypeptides as well as the development of highly potent drugs. Additionally, patients with NAFLD have higher levels of PEDF (Yilmaz *et al.* 2011), which contributes to the non-invasive diagnosis (Yang *et al.* 2015), and the excessive PEDF leads to an elevated susceptibility to intestinal inflammation, which, together with the raised PEDF in patients with IBD during the acute phase, hints at the potential role as a biomarker linking CLD and acute IBD correlation.

**Figure 1:**
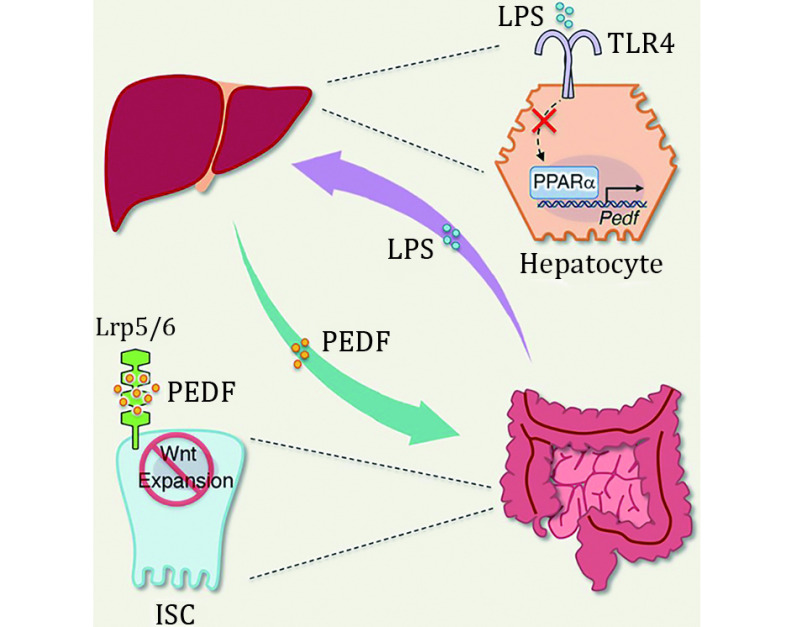
The cellular and molecular mechanisms of the gut–liver axis in calibrating ISC proliferation via PEDF under physiological and inflammatory conditions (Kim *et al.* 2024)

Notably, this study also raises concerning issues. In exploring the role of hepatokines on ISC proliferation, PEDF was screened because of its simultaneous eligibility for Wnt suppressor, liver secretomics, and plasma proteomics, yet other compliant ligands cannot be precluded from influencing ISC stemness. Subsequently, PEDF can act as a ligand for several receptors with various functions, such as due to its immune-related effects, additional splenectomy was required to exclude interfering factors in the present study, whereas PEDF is known to exacerbate the disease through the activation of inflammatory macrophages (Jones *et al.* 2018), further studies are necessary to elucidate the specific features of the different PEDF receptors in relation to intestinal homeostasis. Meanwhile, nor can other DAMPS apart from LPS as TLR ligands be dismissed in the process of microbial danger signals perceived by the liver. Moreover, we also observed that hepatectomy had no significant effect on mice fecal gut microbiota species at the phylum level, however, this still cannot be completely ruled out as a factor due to its geographic specificity of intestinal tracts as well as the possibility of PEDF-mediated dynamic regulation (Koning *et al.* 2023). What's more interesting is that whether PEDF can also indirectly affect ISC stemness by interacting with components of the intestinal stem cell niche, such as the mesenchymal-induced BMP functional gradients (Wu *et al.* 2021), needs to be further investigated. In furtherance, PEDF is also proven to support neural stem cell proliferation (Andreu-Agulló *et al.* 2009; Brook *et al.* 2020), which should draw more attention to whether hepatic PEDF communicates with other organs to influence cell stemness. In conclusion, the study by Kim *et al.* elucidates novel cellular and molecular mechanisms by which the gut–liver axis bidirectionally calibrates intestinal homeostasis and lays the groundwork for future studies of intestinal physiology and tissue repair between health and disease.

## Conflict of interest

Ying Huang, Xinran Wang and Lulu Sun declare that they have no conflict of interest.
